# Observing climate change trends in ocean biogeochemistry: when and where

**DOI:** 10.1111/gcb.13152

**Published:** 2016-01-06

**Authors:** Stephanie A. Henson, Claudie Beaulieu, Richard Lampitt

**Affiliations:** ^1^ National Oceanography CentreEuropean WaySouthamptonSO14 3ZHUK; ^2^ Ocean and Earth SciencesUniversity of SouthamptonEuropean WaySouthamptonSO14 3ZHUK

**Keywords:** attribution, carbon export, chlorophyll concentration, fixed point observatories, monitoring, nitrate, small phytoplankton, sustained observations

## Abstract

Understanding the influence of anthropogenic forcing on the marine biosphere is a high priority. Climate change‐driven trends need to be accurately assessed and detected in a timely manner. As part of the effort towards detection of long‐term trends, a network of ocean observatories and time series stations provide high quality data for a number of key parameters, such as pH, oxygen concentration or primary production (PP). Here, we use an ensemble of global coupled climate models to assess the temporal and spatial scales over which observations of eight biogeochemically relevant variables must be made to robustly detect a long‐term trend. We find that, as a global average, continuous time series are required for between 14 (pH) and 32 (PP) years to distinguish a climate change trend from natural variability. Regional differences are extensive, with low latitudes and the Arctic generally needing shorter time series (<~30 years) to detect trends than other areas. In addition, we quantify the ‘footprint’ of existing and planned time series stations, that is the area over which a station is representative of a broader region. Footprints are generally largest for pH and sea surface temperature, but nevertheless the existing network of observatories only represents 9–15% of the global ocean surface. Our results present a quantitative framework for assessing the adequacy of current and future ocean observing networks for detection and monitoring of climate change‐driven responses in the marine ecosystem.

## Introduction

Ongoing climate change will affect marine ecosystems in a myriad of ways. Increasing atmospheric CO_2_ concentration results in a lowering of ocean pH which may, amongst other effects, impair the viability of calcareous organisms (Doney *et al*., 2012). Warmer temperatures will tend to increase ocean stratification, restricting the supply of nutrients to photosynthetic organisms in the surface waters (Steinacher *et al*., 2010). Warmer waters will also reduce the solubility of oxygen and exchange of subsurface low oxygen waters with the atmosphere, potentially resulting in deoxygenation with subsequent negative effects on marine organisms (Vaquer‐Sunyer & Duarte, 2008). The synergistic effect of these changes is predicted to be an overall decrease in primary production (PP) over most of the global ocean, with the possible exception of the Arctic region (Bopp *et al*., 2013). In addition, in high latitudes the structure of the phytoplankton community is expected to shift away from dominance by large species to smaller pico‐ and nanoplankton as the subtropical gyres expand (Henson *et al*., 2013). In turn, this reduction in large phytoplankton is predicted to result in a decrease in upper ocean carbon export (Doney *et al*., 2014) and hence a reduction in organic carbon sequestration.

Given the critical role that the ocean biosphere plays in regulating Earth's climate (Kwon *et al*., 2009) and in providing the primary protein source for ~15% of the world's population (FAO, 2012), detecting the influence of climate change is clearly a high priority. Rapid detection of the effects of climate change assists in understanding the influence of anthropogenic forcing on the marine ecosystem, which permits more effective decision‐making regarding dependent socioeconomic systems.

As part of the effort to observe and detect long‐term change, a network of open ocean observatories aims to provide long time series of biogeochemical data on both state and rate variables. Previous work has demonstrated that ~30–40 years of continuous data are needed to distinguish a climate change trend in chlorophyll concentration or PP from the background natural variability (Henson *et al*., 2010). Metrics derived from chlorophyll data, for example bloom timing, are not able to reduce the 30–40 year timescale for trend detection (Henson *et al*., 2013) and, in addition, gaps in the time series strongly impact the detection of trends (Beaulieu *et al*., 2013). Nevertheless, some of the longer‐running ocean time series stations are now nearing this predicted threshold for the climate change trend in chlorophyll or PP to become detectable. However, time series stations typically record a variety of parameters beyond chlorophyll or PP; as suggested by Henson *et al*. (2010) the climate change‐driven trend may be more rapidly detectable in other biogeochemical data, a hypothesis which has yet to be tested. Financial and logistical constraints limit the network of time series stations to a relatively small number, mostly close to land to allow regular servicing. As it is unfeasible to fill the ocean with observing stations, an additional important consideration is the size of their ‘footprint’ – or the extent to which a station is representative of a larger region. Large data‐poor regions of the ocean may compromise our ability to detect long‐term trends on large scales. In this study we aim to assess when and where climate change may be detectable with the current monitoring network of open ocean observatories.

These issues are central to several of the ‘10 Commandments’ of climate monitoring, as defined in Karl *et al*. (1995). In particular, there is a need to determine the spatial and temporal resolution of data required to detect climate trends. This will then allow a framework for prioritizing the establishment of new sites in regions that are data‐poor or sensitive to change, and for providing justification for maintaining operation of well‐established time series stations (Henson, 2014). In the analysis presented here we provide quantitative information on both the time and space scales needed for observing climate change effects on a range of ocean biogeochemical variables, which establishes a basis for assessing the adequacy of the current ocean observatory network for climate monitoring.

## Materials and methods

Output from eight Earth System Models (Table S1) forced with the IPCC's ‘business‐as‐usual’ scenario (RCP8.5; Moss *et al*., 2010) for the period 2006–2100 and historical (1860–2005) and control runs (last 100‐year chunk available) was downloaded from the CMIP5 archive (http://pcmdi9.llnl.gov). The variables used were annual average sea surface temperature (SST), surface pH, surface chlorophyll concentration and surface nitrate concentration, annual integrated PP and nondiatom PP, annual total export flux at 100 m depth, and annual average oxygen concentration averaged over the 200–600 m depth range, which encompasses the main thermocline (Gruber, 2011; Bopp *et al*., 2013). Nondiatom PP is used here as a proxy for changes in the relative importance of smaller phytoplankton. These variables were chosen as they are routinely measured at ocean observing stations either autonomously (e.g. temperature, chlorophyll, oxygen) or during regular ship servicing (e.g. PP, export). Additionally, this set of variables is available from a sufficient number of different models to incorporate a quantification of model uncertainty in our analysis.

The linear trend in annual values of the variable for the period of simulation 2006–2100 is calculated using generalized least squares regression:

(1) $$Y_{t}=\mu +\omega X_{t}+N_{t},$$

where *Y*_*t*_ is the time series, *μ* is the intercept, *ω* is the slope (i.e. magnitude of the trend), *X*_*t*_ is the time in years and *N*_*t*_ is the noise, or portion of the data unexplained by the trend. The noise is assumed to be autoregressive of the order 1 so that successive measurements are correlated, as *ϕ *= Corr (*N*_*t*_, *N*_*t*−1_). The number of years of continuous data needed to distinguish a climate change trend from background natural variability is calculated following the method of Tiao *et al*. (1990) and Weatherhead *et al*. (1998). The number of years, n*, required to detect a trend with a probability of detection of 90% and a confidence level of 95% is:

(2) $$n^{*}={\left[\frac{3.3\sigma_{N}}{\omega }\sqrt{\frac{1+\phi }{1-\phi }}\right]}^{2/3},$$

where *σ*_*N*_ is the standard deviation of the noise (residual after trend has been removed). Where less than half of the eight models agree on the sign of the trend, or have nonsignificant trends, those pixels are excluded from further analysis.

The locations of ocean observing stations that include a biogeochemical component were taken from the OceanSites database (http://www.oceansites.org). Here, we limit our analysis to fixed point observatories that are identified in the OceanSites database as currently operational or planned, and which have a biogeochemical component, although they may not measure all the variables explored here (hereinafter these are referred to as BGC‐SOs).

To approximate the region over which samples collected at an observing station are representative (the ‘footprint’), pixels which have similar mean and variability to the time series at the station are identified. First, the time series at each station from a 100‐year section of the model control run for each variable is linearly correlated against every other pixel in the model domain. Where the linear correlation is statistically significant at the 95% level (i.e. *P* < 0.05), and where the pixel is within ±2 standard deviations of the time series mean at the station, the pixel is considered to be representative of conditions at that station. Only regions which are positively correlated with the observing station time series, fall within ±2*σ*, and are contiguous with it are retained for further analysis. In addition, only pixels in which the contiguous patches overlie in at least half of the eight models used here are retained for analysis.

To evaluate the likely realism of the spatial and temporal scales estimated by the model analysis presented here, comparisons to available satellite observations are made. Comparisons of the coefficient of variation and footprint are presented in the Supporting Information. In general, the model‐derived estimates of n* may be underestimates in specific regions such as the equatorial Pacific for SST and coastal upwelling regions for chlorophyll, as observed variability tends to be larger than modelled variability (see Supporting Information).

Finally, a note on terminology: here we use the term ‘natural variability’ to indicate interannual, decadal, or multidecadal variability forced by oscillatory or transient conditions (e.g. El Niño‐Southern Oscillation, North Atlantic Oscillation etc.). In contrast, ‘trends’ is used to refer to long‐term (multidecadal or longer) changes driven by persistent anomalous forcing (in this case global climate change).

## Results

### Climate change trends in ocean biogeochemistry

The predicted trends from 2006 to 2100 in the eight variables used here are plotted in Fig. S1. As expected, pH decreases and SST increases throughout the world's oceans. The Arctic experiences the most rapid decrease in pH due to the retreat of sea ice which increases absorption of atmospheric CO_2_ (McNeil & Matear, 2007). Strongest warming trends occur in the Northwest Pacific and in the tropics, with slower warming in the North Atlantic and Southern Ocean, coinciding with regions of deep water formation. Thermocline oxygen content declines almost everywhere due to warming‐related decreases in the solubility of gases and reduced ventilation. The exception is in the equatorial regions which are currently low oxygen zones. Here, it seems that the decrease in exported organic carbon (and hence presumably decreased remineralization) outweighs the expected decline in oxygen content due to warming (Deutsch *et al*., 2014). Surface nitrate concentration decreases almost everywhere, as may be expected from reduced mixing. The exception is at the edges of the oligotrophic gyres, a pattern which appears only in the IPSL family of models and may be driven by decreased biomass of nanoplankton due to increased grazing pressure (Laufkötter *et al*., 2015). PP, nondiatom PP and export all have similar patterns, with decreasing trends throughout low latitudes likely due to increased stratification, and increasing trends in the Southern Ocean and Arctic, which are particularly pronounced in currently ice‐covered regions. Interestingly, chlorophyll shows the same patterns as PP except in the Arctic where chlorophyll decreases but PP increases. This is accompanied by a very strong increasing trend in nondiatom PP which suggests that diatoms are replaced by smaller phytoplankton with a lower chlorophyll : carbon ratio.

### Length of time series required to detect trends

**Figure 1:**
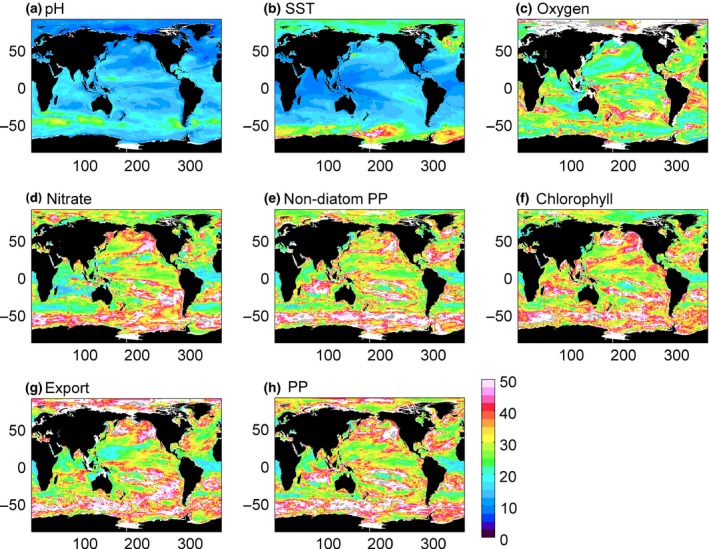
Number of years of data needed to distinguish a climate change‐driven trend from natural variability for (a) pH, (b) SST, (c) oxygen, (d) nitrate, (e) nondiatom PP, (f) chlorophyll, (g) export and (h) PP. White areas are where less than half of the eight models used here agree on the sign of the trend. Grey dots indicate where the climate change trend does not exceed the natural variability in the timeframe of the simulations (95 years).

For a trend to be rapidly detectable, the ratio of the signal (i.e. the trend magnitude) to the noise (i.e. the variability) needs to be high. The number of years of data needed to detect a trend (n*) in biogeochemical variables is shown in Fig. 1. The natural variability, here defined as the standard deviation of the residuals (see Materials and methods), is presented in Fig. S2. In many variables, n* is shorter at low than at high latitudes. This arises because at low latitudes trends are strong but variability tends to be smaller than at high latitudes, and therefore fewer years of data are needed to detect a long‐term trend. An example of how the noise and signal interact in estimating n* can be found in the Northeast Pacific oxygen content, where low noise and a strong decreasing trend result in an estimated ~25 years of data needed to detect a trend. A scenario at the other extreme is nitrate concentration in the Southern Ocean which has weak noise, but also a weak trend, resulting in larger n* (>40 years).

The climate change‐driven trends in pH and SST are most rapidly distinguished from natural variability, requiring just 13.9 and 15.5 years of data, respectively (global median values; Table 1). Strong trends in response to anthropogenic forcing and, in the case of pH, low natural variability result in rapidly detectable trends, in particular in low latitude regions. For SST, between 20 and 50 years of data are needed to detect a trend in the Southern Ocean and high latitude North Atlantic where warming trends are smallest.

**Table 1:** Global median n* and footprint size

	PP (integrated)	SST (surface)	pH (surface)	Oxygen (200–600 m)	Nitrate (surface)	Chlorophyll (surface)	Export (100 m)	Nondiatom PP (integrated)
n*, years	32.3	15.5	13.9	26.3	29.7	31.5	32.0	30.9
Footprint, 10^6^ km^2^ (% ocean covered)	42.9 (11)	48.6 (13)	58.5 (15)	33.4 (9)	51.1 (13)	47.9 (12)	42.7 (11)	42.4 (11)

Global median value of number of years of data required to detect a climate change‐driven trend above background variability (n*) for eight biogeochemical variables. Global coverage for each variable (10^6^ km^2^) with percentage of ocean surface covered given in brackets. Full information on n* and footprint size for all BGC‐SO sites considered can be found in Supporting Information (Tables S2 and S3, respectively).

Aside from SST and pH, all other biogeochemical variables require, as a global average, 26–33 years of data to detect a trend (Table 1). Of these, trends in oxygen are most rapidly detectable, particularly in the North Pacific and eastern Indian Ocean (15–20 years) where strong decreasing trends in oxygen content are predicted (Fig. S1). Nitrate, chlorophyll, PP, nondiatom PP and export all have a minimum in n* (<20 years) in the equatorial Atlantic and Benguela region. Trends in nitrate and both total and nondiatom PP are also rapidly detectable (n* < 25 years) in the Atlantic and Indian sectors of the Southern Ocean, at approximately the latitude of the Antarctic Circumpolar Current.

Strong decreasing trends in nitrate and chlorophyll in the Atlantic and east Pacific sectors of the Arctic result in rapidly detectable trends (n* < 25 years). Nevertheless, PP, nondiatom PP and export all have strong increasing trends, although this doesn't necessarily result in shorter n* due to high natural variability (Fig. S2) in these regions.

**Figure 2:**
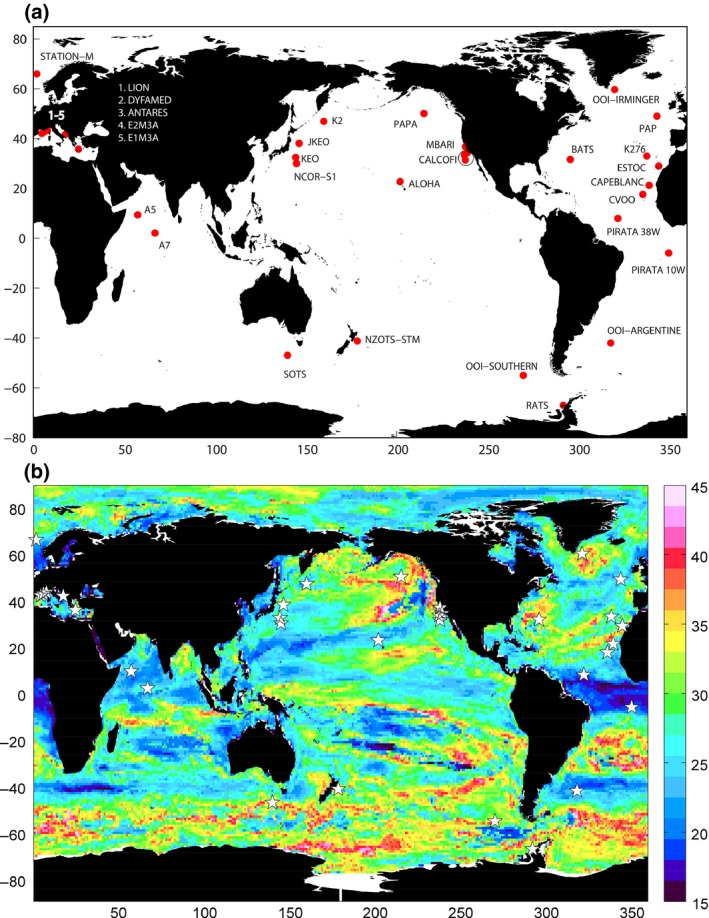
(a) Map of ocean time series stations with a biogeochemical component (referred to as BGC‐SOs in the text). The map includes only currently operating and planned stations, according to http://www.oceansites.org. (b) Median value of n* (number of years of data required to detect a climate change‐driven trend above background variability) for all eight variables considered here. Plotted is the multivariable median of the multimodel medians shown in Figure 1. White stars mark locations of BGC‐SOs.

As an indication of the overall rapidity with which climate change trends may be detectable in a range of parameters, the median n* calculated across all eight variables is plotted in Fig. 2. The regions where detecting trends requires the shortest time series of observations (<20 years) are the equatorial Atlantic and Benguela upwelling, in which all variables have relatively short n* (Fig. 1) due to strong trends and weak natural variability. With 25 years of data, trends are detectable in large parts of the Indian Ocean, particularly the Arabian Sea, and parts of the South and North Pacific gyres, plus a band at 40°S. Long time series of data (>40 years) are needed to detect trends in parts of the Southern Ocean, Northeast Pacific, South Atlantic gyre and northern North Atlantic. In the case of the North Atlantic (Irminger Basin), all variables except pH require very long time series to detect a trend. For the other regions, n* is long in all variables except pH, SST and oxygen.

Also plotted in Fig. 2 are the positions of 33 planned and existing ocean observing stations which have a significant biogeochemical component. In some cases (Table S2), these stations are located in regions where climate change should be relatively rapidly detectable, for example at ALOHA (ranging from ~13 years for SST to ~29 years for chlorophyll concentration) or the PIRATA stations in the equatorial Atlantic (from ~11 years for SST to ~26 years for oxygen at the southerly station). At other stations, considerably longer time series would be needed to distinguish a climate change trend, for example at BATS (ranging from ~11 years for SST, ~47 years for export flux, to undetectable in a 95 year record for PP) or the Southern Ocean Time Series station off Tasmania (from ~20 years in SST to ~46 years in chlorophyll, to undetectable in export flux).

### Spatial footprints of current ocean observatory network

Ocean observing stations are typically single point locations, although in some cases arrays of sampling may be carried out, such as the CALCOFI (southern California) grid covered by regular cruises. Here, we assess whether the current network of ocean observing stations provides adequate coverage of ocean conditions to determine a climate change trend. The spatial area wherein each observing station is statistically similar, in terms of its mean and variability, with surrounding conditions (see Materials and methods) provides an indication of each station's ‘footprint’, that is where single‐point observing stations may be considered representative of conditions in a larger area.

**Figure 3:**
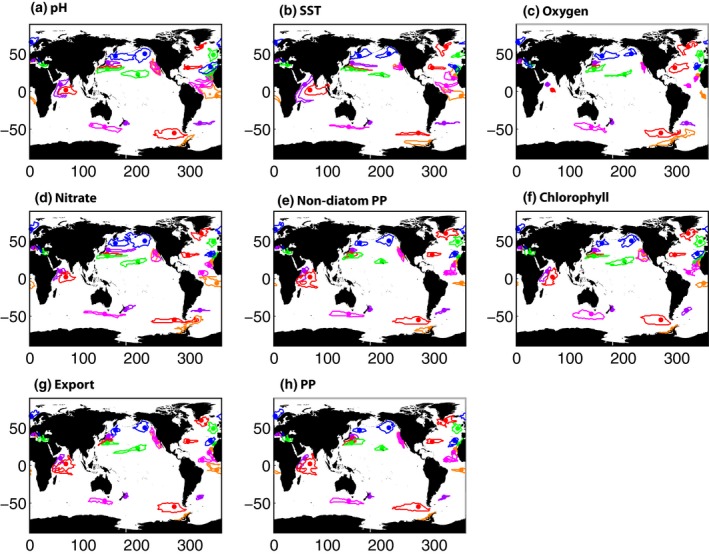
Spatial footprint maps calculated for (a) pH, (b) SST, (c) oxygen, (d) nitrate, (e) nondiatom PP, (f) chlorophyll, (g) export and (h) PP. Each BGC‐SO is represented by a dot with its corresponding footprint contoured in the same colour.

Figure 3 shows the footprint size at selected BGC‐SOs for all eight variables. Generally, the stations are representative of biogeochemical properties in fairly localized areas, ~1.1 × 10^6^ km^2^ on average. Stations in the Arabian Sea, North Pacific and Southern Ocean are representative of the largest areas (e.g. ~4.85 × 10^6^ km^2^ for A7, median for all eight variables), whilst those in the Mediterranean are representative of the smallest regions (e.g. ~0.18 × 10^6^ km^2^ for LION, median for all eight variables). The variables with the largest footprints are SST, pH and nitrate (Fig. 3), but nevertheless the current network of BGC‐SOs is still only representative of 13–15% of the ocean (Table 1). A second group of variables (chlorophyll, PP, nondiatom PP and export) are the biological response to changing physical and chemical conditions and have smaller footprints so that BGC‐SOs represent 11–12% of the ocean. Oxygen concentration has the smallest footprint, with only 9% of the ocean covered by existing BGC‐SOs. For all variables, any region not in close proximity to a BGC‐SO is unrepresented, mainly in the Southern Hemisphere and Arctic. A table with full listing of areal extent represented by each BGC‐SO and variable is in Table S3.

Spatial patterns of coverage for all variables are shown in Fig. S3 which plots the number of BGC‐SOs that are representative of each 1 × 1° pixel, that is the regions of overlap in Fig. 3. Some ocean regions are very well‐represented by multiple BGC‐SOs, such as the west coast of the USA. Much of the North and equatorial Atlantic, North Pacific, western Indian Ocean and Mediterranean are covered by existing BGC‐SOs, but vast tracts of the Southern Hemisphere are not.

## Discussion

### Limitations

The results presented here are dependent on the ability of the models used to represent the observed natural variability. Global‐scale information on variability derived from satellite data is only available for SST and chlorophyll concentration (and derived products, such as PP). Overall, the models represent the natural variability in SST very well, although it is underestimated in the Arctic (Fig. S4). For chlorophyll, observed variability is generally larger than modelled in the coastal and polar regions (Fig. S5). Typically, modelled variability is lower than observed variability, particularly in biogeochemical parameters (e.g. Cadule *et al*., 2010; Laepple & Huybers, 2014). Generally, we find a similar pattern, although with some exceptions such as chlorophyll in the oligotrophic gyres in the HadGEM models (see Fig. S5). Increased levels of variability would result in weaker signal to noise ratio, and thus longer n*. Note also that the observations do not represent purely natural variability as they contain responses to both natural and anthropogenic forcing. The modelled spatial footprints are similar to those calculated from observed SST and chlorophyll (Fig. S6, compare to Fig. 3b, f). For SST, the pattern and shape of the footprints are very similar in observations and models, although they are slightly larger in the observations so that 18% of the ocean is covered by BGC‐SOs (13% for models). In chlorophyll, results are mixed with the size, shape and isotropy of the footprints being similar in observations and models for some BGC‐SOs, for example BATS and PAP, but not in others, for example OOI‐Argentine. Both observations and models suggest that 12% of the ocean is represented by BGC‐SOs for chlorophyll, although the distribution of coverage is slightly different.

The size and shape of the footprints is also dependent on our definition of ‘representative’, which takes into account the mean and variability of time series. For some studies, other metrics of representativeness will be more appropriate and the size of the footprints would thus change accordingly. Additionally, we test repeatedly for statistical significance. At the 5% critical level chosen here, 1 in 20 regressions will produce a ‘false positive’ (i.e. significant when no correlation is actually present), and therefore footprints may in reality be smaller than shown here. Finally, the relatively coarse resolution of the models excludes (sub)‐mesoscale variability which will act to reduce the size of the footprints.

In our analysis, we test only for linear trends by using a generalized least squares approach. In reality, trends may be nonlinear. We tested whether our linear fits are reasonable by verifying the underlying assumptions that the residuals are AR(1) and normally distributed with a constant variance and found that the majority (~66%) of calculated trends meet these assumptions. Furthermore, even though the linear trends estimated using generalized least squares seem reasonable to estimate n* in most cases, using different trend detection techniques based on nonparametric statistics or Bayesian approaches (e.g. Chandler & Scott, 2011) may result in different estimates of n*.

Finally, the coarse spatial resolution (1°) of the models (i.e. mesoscale variability is not simulated) and the potential underestimation of temporal variability in the models imply that our results are likely to be a ‘best case’ scenario. In reality, the footprints are likely to be smaller than estimated here, due to unresolved mesoscale variability; similarly, n* is likely to be longer than estimated here, due to unresolved temporal variability.

### Time and space scales of observation

The analysis presented here quantifies the time and space scales over which biogeochemical observations need to be made to detect climate change trends, and makes a preliminary assessment of whether the current BGC‐SO network meets those requirements. In general, SST and pH are most rapidly detectable (n* ~15 years). These variables respond directly to increasing atmospheric CO_2_ concentration, resulting in strong trends. In addition, natural variability is relatively low in SST and pH, particularly in low latitudes. Note however that pH can be very variable in coastal regions (Duarte *et al*., 2013) which are not resolved in these global models.

In keeping with our previous work (Henson *et al*., 2010), we find that chlorophyll and PP require longer time series to detect trends (n* ~32 years). A key question arising from the earlier work was whether climate change‐driven trends could be more rapidly detectable in other biogeochemical variables. Here, we find that the answer is generally ‘no’ (except for pH), with oxygen and nitrate concentration, export and nondiatom PP all requiring time series >25 years in length (global median; Table 1). There are however clear regional differences, with the equatorial Atlantic, Benguela and Arabian Sea requiring fewer years of data to distinguish a trend. In contrast, in parts of the Southern Ocean and Northeast Pacific, detecting trends requires very long records and in some cases changes are nondetectable in the 95 year timescale of the simulations used here. These results are in contrast with the hypothesis that ecosystems can ‘integrate’ the environmental conditions that they experience, improving the signal to noise ratio and permitting detection of weak climate change‐driven trends (Taylor *et al*., 2002). Instead we find that detection time is shorter for environmental forcing factors than for the ecosystem response.

According to our analysis, some BGC‐SOs have been in operation sufficiently long that climate change trends in some variables should be detectable (Table S2). For example, ALOHA was established in 1988 (~27 years ago at the time of writing) and so climate change trends may now be detectable in their records in all parameters except chlorophyll concentration. Indeed, trends have been identified in pH, PP, SST, chlorophyll concentration and nitrate at ALOHA (Saba *et al*., 2010; Kim *et al*., 2014). The presence of a statistically significant trend in chlorophyll at ALOHA is inconsistent with our estimate of n*, implying that the observed trend should not yet be ascribed to climate change (or that the modelled n* is an overestimate, see Limitations section). Other stations need to be in operation for some years more, for example PAP where, although trends in pH may be detectable after 14 years of operation, other variables require ~25 years of data. With some exceptions, the current network of BGC‐SOs has not been in operation sufficiently long to detect climate change‐driven trends.

An analysis of the SST and pH data collected at BATS illustrates how the n* estimates presented in Table S2 and Fig. 1 can be applied to *in situ* time series. At BATS, our analysis suggests that ~15 years of data are needed to distinguish a long‐term trend in pH from background variability (Table S2). We split the observed pH time series (Bates *et al*., 2012 and N. Bates, personal communication) into overlapping 10 and 15 year chunks and tested for trends by applying Eqn (1). Time series as short as 10 years evince statistically significant trends for most periods, however our analysis suggests that, with an estimated n* of 15 years, it would be ill‐advised to unequivocally attribute the trend in a 10 year time series to climate change. For 15 year time series, all periods show a statistically significant trend which is likely a genuine long‐term trend. For the whole time series of 30 years, the trend is −0.002 units, which is statistically significant at the 95% level. As the length of the complete time series far exceeds the n* suggested by our analysis, this trend is highly likely to be climate change‐driven.

For the SST time series at BATS, we find that n* is ~11.5 years (Table S2). Splitting the time series into 10 year pieces, a statistically significant trend is found for the period 1987–1996 (but not for other 10 year chunks). Our analysis suggests that this is unlikely to be a long‐term trend as the time series is shorter than the estimated n*. Indeed, increasing the length of the time series chunks to 11 years yields the result that the trend in the period 1987–1997 is only just statistically significant at the 95% level. Adding another year of data (forming 12 year pieces) results in the trend for the period 1987–1998 becoming nonsignificant. This result matches exactly with our analysis: that any apparent trend in SST in a dataset shorter than n* (in this case 11.5 years) is unlikely to be due to climate change forcing, but instead is likely to be an artefact associated with natural variability dominating the short time series. For the entire BATS SST time series, there is no statistically significant trend. As the dataset is substantially longer than our estimated n*, it is likely that if a climate change‐driven trend were present it would be detectable, suggesting that anthropogenic forcing of SST at BATS is not yet distinguishable from natural variability.

Complementary to estimates of the timescales over which observations need to be made is the consideration of the spatial scales required to ensure adequate representation of ocean conditions. Our results demonstrate that the spatial footprint for SST, pH and nitrate is larger than for biogeochemical variables such as PP. As such, the current network of BGC‐SOs are representative of ~15% of the ocean for pH, but only 9% for thermocline oxygen concentration (Table 1). For some of the variables considered here there are plentiful alternative sources of data, such as satellite‐derived SST and chlorophyll which ensure global‐scale coverage of surface conditions ensuring that data availability is unlikely to limit trend detection. In addition, platforms such as Argo and bio‐Argo floats, gliders and other autonomous vehicles can provide depth‐resolved data on temperature, chlorophyll, oxygen etc. Therefore for these variables, our calculations underestimate the proportion of ocean represented by current observations. However, variables such as carbon export or phytoplankton functional type‐specific PP are currently not routinely derived from autonomous platforms, so that ship‐serviced BGC‐SOs are the primary source of data.

The current observing network results in substantial regions of the ocean which are not represented by any BGC‐SO. The Arctic and the majority of the Southern Hemisphere are particularly poorly covered. Some of these areas overlap with regions where trends are likely to be rapidly detectable (Fig. 2b), implying that timely detection of climate change‐driven trends in ocean biogeochemistry may be jeopardized by the sparsity of the observations in these areas.

Other regions, notably the west coast of the USA, are represented by multiple BGC‐SOs. One may ask therefore whether some stations could be cut without loss of information for climate change detection. However, it should be noted that the global models used here are relatively coarse resolution (1 × 1°). In addition, this analysis focuses only on the requirements for climate change detection, whereas BGC‐SOs serve many other purposes. Spatial variability will occur on scales smaller than the model resolution, which may be relevant for the primary goals of a BGC‐SO. For example in the case of CALCOFI, a primary goal is fisheries assessment, for which higher spatial resolution sampling is likely to be necessary than for climate trend detection.

### Implications for ocean observatories

Our results allow an initial assessment of the adequacy of the current BGC‐SO network for climate change trend detection, in terms of space and time scale considerations. In many cases, long running SOs are close to having sufficiently long time series to distinguish climate change‐driven trends from background natural variability, for example ALOHA (Table S2). However, these well‐established SOs provide only limited spatial coverage (Fig. 3). Several of the BGC‐SOs considered here are relatively new or still in the planning stages and so, although some of them fill in gaps in the spatial coverage of the network, they may require decades more data before a climate change trend can be detected.

**Figure 4:**
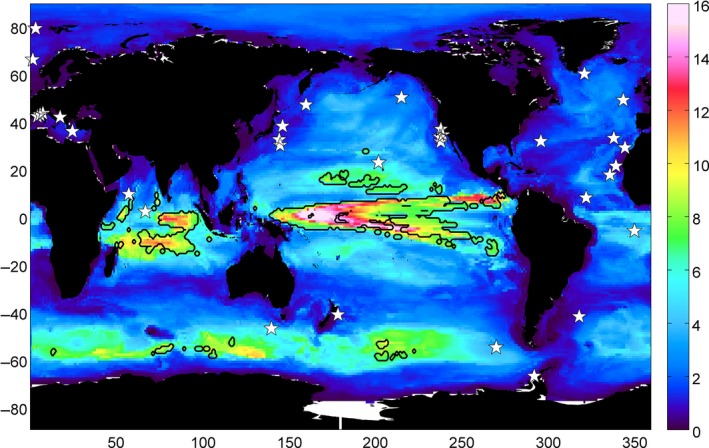
Size of footprint (10^6^ km^2^) for chlorophyll time series calculated at every grid point, where the footprint is defined as pixels that have similar mean and variability (see Materials and methods). Black contour marks where the footprint is relatively large (>7 × 10^6^ km^2^) and n* is relatively short (<35 years). White stars mark locations of BGC‐SOs.

If the opportunity arose to design a new ocean observatory, with the primary goal of detecting climate change trends, the optimal location would be in a region of large spatial length scales and rapidly detectable trends (ignoring logistical issues). As an example, the size of the footprint for chlorophyll concentration calculated at every grid point (in the same way as for individual BGC‐SOs, see Materials and methods) is plotted in Fig. 4. Largest footprints for chlorophyll are located in the equatorial Pacific and Indian Oceans, and parts of the Southern Ocean. In the case of the equatorial regions, these also overlap with areas of relatively short n* (<35 years), marked with a black contour in Fig. 4. None of the existing BGC‐SOs are located within these optimal trend detection regions.

Although existing BGC‐SOs may not necessarily be in an ideal location if the primary aim is detecting climate change, for the well‐established stations little is to be gained from relocating them. Generally, n* is >20 years (except for SST and pH), so if >20 years of data have already been collected, then in most cases more than half the required time series to detect climate change is already in hand (and in some cases much more than half). Importantly, some BGC‐SOs do not have climate change detection as a primary goal, focusing instead on process understanding. In these cases, the discussion presented here of time and space scales is of less relevance.

Our results suggest that the current network of BGC‐SOs is, in some cases, adequate to assess climate change trends at the local scale. Some BGC‐SOs may already have, or will soon have, sufficiently long time series to detect climate change‐driven trends. Care, however, needs to be taken when calculating trends. Autocorrelation, which is prevalent in geophysical time series, can lead to the detection of spurious trends if not accounted for, particularly when using short time series (Wunsch, 1999). In addition, any ‘interventions’ in the time series, such as due to changes in sampling methodology or instrumentation, gaps in the dataset, relocation of sampling site etc., will tend to increase the number of years of data needed to detect a trend as the intervention effect must then be estimated and accounted for (Beaulieu *et al*., 2013). Finally, careful choice of the appropriate statistical model to fit to the data must be made as trends may not be linear.

At the global scale, the existing BGC‐SO network is representative of 9–15% of the ocean (Table 1). Clearly in order for large‐scale biogeochemical trends to be characterized, the BGC‐SOs must be augmented with additional data. Our analysis makes clear that continued satellite‐derived observations are essential due to their global coverage, but also that ongoing support of existing BGC‐SOs and more rapid development of autonomous methods, including the bio‐Argo network, would be beneficial. Currently ~200 bio‐Argo floats have been deployed, emphasizing that concerted international effort on the scale of the original Argo programme is required to achieve coverage on time and space scales suitable for climate change detection. The biological and biogeochemical properties that can be measured by bio‐Argo floats and other autonomous methods are currently limited, in particular when sensors are large or have high power requirements, or where sample collection is necessary. The network of ocean observatories will therefore still need to be maintained into the future. However, targeted deployment strategies could be used to sample regions with particularly poor data coverage, large spatial footprints and short detection times. The preliminary assessment carried out here could be developed into a full Observing System Simulation Experiment (e.g. Majkut *et al*., 2014) to strategically optimize the observing network to ensure maximum effect for minimum effort.

## Supporting information


**Appendix S1.** Comparison of model output and satellite observations.
**Table S1.** Models used in the analysis.
**Table S2.** Number of years of data required to detect a climate change‐driven trend above background variability for eight biogeochemical variables at BGC‐SO sites.
**Table S3.** Size of footprints for all BGC‐SOs (10^6^ km^2^) for all eight variables.
**Figure S1.** Trends in all eight variables for the period 2006–2100.
**Figure S2.** Noise (i.e. natural variability) used in the calculation of n* normalized to the model mean.
**Figure S3.** Number of BGC‐SOs which have overlapping footprints.
**Figure S4.** Comparison of interannual variability in models and observations for SST.
**Figure S5.** Comparison of interannual variability in models and observations for chlorophyll.
**Figure S6.** Spatial footprints calculated for SST and chlorophyll using satellite observations.
