# Supplementary material for: Observing climate change trends in ocean biogeochemistry: when and where
Source: Glob Chang Biol. 2016 Jan 6;22(4):1561–71. doi: 10.1111/gcb.13152 (PMC4785610; doi:10.1111/gcb.13152)
Supplement: Supplementary file 1 — Appendix S1. Comparison of model output and satellite observations. Table S1. Models used in the analysis. Table S2. Number of years of data required to detect a climate change‐driven trend above background variability for eight biogeochemical variables at BGC‐SO sites. Table S3. Size of footprints for all BGC‐SOs (106 km2) for all eight variables. Figure S1. Trends in all eight variables for the period 2006–2100. Figure S2. Noise (i.e. natural variability) used in the calculation of n* normalized to the model mean. Figure S3. Number of BGC‐SOs which have overlapping footprints. Figure S4. Comparison of interannual variability in models and observations for SST. Figure S5. Comparison of interannual variability in models and observations for chlorophyll. Figure S6. Spatial footprints calculated for SST and chlorophyll using satellite observations. [file GCB-22-1561-s001.docx]

**Supporting Information**

**Comparison of model output and satellite observations**

As a first order check of the models’ representation of spatial and temporal variability, the output is compared to satellite observations of SST and surface chlorophyll. Only satellite data can provide the global, multi-year time series of data needed to check model fidelity and so we are restricted to using only SST and chlorophyll. Two metrics are compared in observations and models: the degree of interannual variability (or ‘noise’) and the size and shape of the spatial footprints.

The interannual variability in modelled and observed SST and chlorophyll is shown in Figures S4 and S5, respectively. The variability or ‘noise’ is a key component of *n**, along with the trend. If the modelled noise is less than the observed, that suggests that the estimated *n** is likely to be an underestimate, and vice versa. The modelled interannual variability in SST is very similar to the observed in most regions, except the Arctic Ocean (Figure S4) where the models generally underestimate the variability in SST. For chlorophyll, observed variability is larger than modelled in some coastal and polar regions (Figure S5); however, some models overestimate variability in the oligotrophic gyres. Uncertainty in the magnitude of natural variability can affect estimates of *n**. As an example for SST in the equatorial Pacific, an underestimate of the noise by a factor of 2, given typical trends (~ 0.03 °C yr^-1^) and autocorrelation ( ~ 0.01) values, would result in *n** decreasing from ~ 25 to 15 years. The model natural variability used to calculate *n** is shown in Figure S2.

The spatial footprints calculated from observed SST and chlorophyll are plotted in Figure S6 and should be compared to the model counterparts in Figures 3b and 3f, respectively. Note however that the observed footprints are calculated over a period of 30 years (SST) and 17 years (chlorophyll), whereas the model footprints are calculated for a 100 year period. For SST, the pattern and shape of the footprints are very similar in observations and models, although they are slightly larger in the observations so that 18 % of the ocean is covered by BGC-SOs (13 % for models). In chlorophyll, results are mixed with the size, shape and isotropy of the footprints being similar in observations and models for some BGC-SOs, e.g. BATS and PAP, but not in others, e.g. OOI-Argentine. Both observations and models suggest that 12 % of the ocean is represented by BGC-SOs for chlorophyll, although the distribution of coverage is slightly different.

Another important point to note regarding the model representation of natural variability is that the models were not run in hindcast mode, where the ocean is forced by observed atmospheric conditions, which allows a direct year-to-year comparison with observations (e.g. a strong El Niño event should occur in 1997/98). Instead, these ocean models are coupled to atmospheric models which create their own variability which should be statistically similar to observations over long time periods, although specific events may not occur in the same year as in reality (e.g. an El Niño should occur every ~ 3-7 years, but not necessarily coinciding with observed El Niño events). This may also lead to an apparent underestimation of the observed interannual variability.

**Additional References**

Collins WJ, Bellouin N, Doutriaux-Boucher M et al. (2011) Development and evaluation of an Earth-System model-HadGEM2, Geoscientific Model Development. **4**, 1051-1075.

Dunne JP, John JG, Adcroft AJ et al. (2012) GFDL's ESM2 Global Coupled Climate-Carbon Earth System Models. Part I: Physical Formulation and Baseline Simulation Characteristics, Journal of Climate. **25**, 6646-6665.

Dunne JP, John JG, Shevliakova E et al. (2013) GFDL's ESM2 Global Coupled Climate-Carbon Earth System Models. Part II: Carbon System Formulation and Baseline Simulation Characteristics, Journal of Climate. **26**, 2247-2267.

Moore JK, Doney SC and Lindsay K (2004) Upper ocean ecosystem dynamics and iron cycling in a global three-dimensional model, Global Biogeochemical Cycles, **18**, doi:10.1029/2004gb002220.

Seferian R, Bopp L, Gehlen M et al. (2013) Skill assessment of three earth system models with common marine biogeochemistry, Climate Dynamics. **40**, 2549-2573.

| **Modeling Center (or Group)** | **Institute ID** | **Model Name(s)** | **Reference** |
| --- | --- | --- | --- |
| Community Earth System Model Contributors | NSF-DOE-NCAR | CESM1(BGC) | Moore et al. 2004 |
| NOAA Geophysical Fluid Dynamics Laboratory | NOAA GFDL | GFDL-ESM2G  GFDL-ESM2M | Dunne et al. 2012, 2013 |
| Met Office Hadley Centre (additional HadGEM2-ES realizations contributed by Instituto Nacional de Pesquisas Espaciais) | MOHC  (additional realizations by INPE) | HadGEM2-CC  HadGEM2-ES | Collins et al. 2011 |
| Institut Pierre-Simon Laplace | IPSL | IPSL-CM5A-LR  IPSL-CM5A-MR  IPSL-CM5B-LR | Seferian et al. 2013 |

**Table S1:** Models used in this analysis. Control, historical and forced runs (RCP8.5) were downloaded from the IPCC CMIP5 archive (http://pcmdi9.llnl.gov/).

| **n^*^, years** | **PP**  **(integrated)** | **SST**  **(surface)** | **pH**  **(surface)** | **Oxygen**  **(200-600 m)** | **Nitrate**  **(surface)** | **Chlorophyll**  **(surface)** | **Export**  **(100 m)** | **Non-diatom PP**  **(integrated)** |
| --- | --- | --- | --- | --- | --- | --- | --- | --- |
| BATS | n/a (-) | 11.4 (2.7) | 15.1 (4.6) | 27.0 (10.8) | 28.4 (6.5) | 41.5 (13.4) | 47.4 (12.2) | 44.7 (4.5) |
| CAPEBLANC | 29.7 (3.5) | 13.8 (1.2) | 16.7 (5.3) | 32.4 (9.2) | 31.6 (6.3) | 26.9 (6.3) | 25.6 (8.3) | 31.2 (5.0) |
| CVOO | 28.6 (6.6) | 11.9 (1.5) | 17.0 (7.2) | 27.2 (16.0) | 27.8 (8.9) | 36.5 (8.6) | 22.0 (10.2) | 35.6 (8.5) |
| ESTOC | 26.9 (7.7) | 18.6 (2.1) | 11.3 (4.9) | 48.9 (14.1) | 25.8 (10.6) | n/a (-) | 29.4 (3.3) | 30.1 (10.1) |
| HAUSGARTEN | 39.1 (7.8) | 28.9 (5.9) | 10.2 (3.2) | 21.0 (20.6) | 28.8 (12.7) | 25.3 (11.5) | n/a (-) | 29.6 (7.7) |
| K276 | 30.0 (11.8) | 13.9 (8.0) | 12.2 (4.5) | 29.6 (15.3) | 22.6 (17.7) | 27.1 (8.0) | 37.8 (11.7) | n/a (-) |
| OOI-ARGENTINE | 28.7 (6.6) | 14.8 (3.1) | 13.8 (4.4) | 24.8 (11.8) | 15.7 (11.4) | 31.5 (12.0) | 33.8 (8.6) | 23.6 (6.2) |
| OOI-IRMINGER | 43.7 (6.8) | 27.6 (9.4) | 8.6 (3.8) | 34.5 (10.4) | n/a (-) | 49.5 (14.6) | 33.8 (14.5) | n/a (-) |
| PIRATA 10W | 18.0 (4.7) | 11.0 (1.9) | 16.6 (5.7) | 25.5 (15.0) | 20.2 (7.3) | 21.8 (13.0) | 19.1 (7.3) | 20.5 (6.8) |
| PIRATA 38W | 22.5 (14.2) | 11.1 (2.1) | 16.4 (6.3) | 21.3 (11.7) | 17.4 (13.7) | 36.7 (16.8) | 21.5 (13.2) | 23.1 (12.9) |
| PAP | 25.9 (6.9) | 21.5 (6.8) | 8.6 (7.8) | 29.5 (11.2) | 27.9 (9.4) | 24.6 (5.4) | 25.1 (11.7) | 22.7 (11.0) |
| MIKE | 29.1 (12.2) | 18.2 (14.9) | 11.7 (3.7) | 21.4 (3.8) | 24.5 (9.1) | 27.4 (3.0) | 25.9 (14.5) | 35.0 (8.3) |
| A5 | 24.3 (13.0) | 9.9 (1.0) | 18.4 (4.2) | 26.7 (9.2) | 22.2 (5.9) | 26.2 (5.4) | 22.5 (4.5) | 24.4 (14.4) |
| A7 | 27.9 (9.9) | 9.4 (1.3) | 16.4 (2.1) | 23.8 (11.8) | 32.9 (16.7) | 32.3 (11.8) | 26.3 (10.6) | 28.2 (10.0) |
| ANTARES | n/a (-) | 12.1 (1.5) | 11.8 (2.3) | 37.0 (14.6) | 24.9 (13.8) | 42.5 (13.0) | n/a (-) | 25.5 (7.4) |
| DYFAMED | 34.2 (12.0) | 12.4 (1.3) | 11.4 (2.0) | 34.6 (11.7) | 25.6 (11.3) | n/a (-) | n/a (-) | 27.1 (13.3) |
| E1M3A | 36.5 (11.5) | 10.1 (3.0) | 13.1 (0.7) | 16.0 (17.9) | 36.3 (14.8) | 37.5 (8.4) | 38.7 (8.0) | 30.3 (5.0) |
| E2M3A | 28.9 (11.9) | 10.3 (3.1) | 12.4 (2.3) | 15.1 (11.2) | 36.9 (11.0) | 37.8 (19.3) | 36.2 (17.2) | 23.1 (2.6) |
| LION | n/a (-) | 12.5 (1.3) | 12.8 (2.1) | 31.6 (12.7) | 28.8 (16.7) | n/a (-) | 43.7 (13.4) | 27.3 (13.1) |
| CALCOFI-080-055 | n/a (-) | 15.7 (1.8) | 12.8 (1.5) | 21.2 (18.1) | 37.9 (6.0) | 36.4 (9.7) | 32.7 (9.1) | 25.5 (14.1) |
| CALCOFI-080-080 | 28.1 (12.7) | 15.0 (4.6) | 12.9 (2.0) | 21.2 (5.8) | 28.7 (10.1) | 27.9 (8.7) | 29.5 (2.0) | 30.6 (12.9) |
| CALCOFI-090-090 | 23.5 (8.9) | 14.2 (4.5) | 12.8 (2.0) | 25.0 (11.6) | 31.1 (6.7) | 31.3 (4.7) | 29.3 (12.1) | 31.1 (10.9) |
| ALOHA | 25.6 (12.0) | 13.3 (3.3) | 13.7 (2.4) | 24.2 (4.4) | 23.3 (18.0) | 29.1 (9.3) | 19.4 (13.4) | 25.8 (12.7) |
| K2 | 43.2 (13.2) | 11.4 (4.6) | 11.5 (1.9) | 22.7 (10.4) | 29.0 (10.3) | n/a (-) | n/a (-) | 23.4 (4.9) |
| JKEO | 26.4 (6.2) | 19.4 (5.3) | 13.8 (3.9) | 29.5 (15.0) | 23.6 (7.5) | 25.3 (7.9) | 30.8 (10.5) | 33.4 (8.8) |
| KEO | 42.3 (6.6) | 13.9 (4.0) | 16.6 (3.6) | 26.2 (6.6) | 30.6 (5.1) | 26.1 (8.6) | 34.4 (12.7) | 44.5 (8.1) |
| MBARI-M0 | n/a (-) | 15.1 (2.7) | 12.2 (1.3) | 23.5 (21.9) | 34.4 (11.5) | 37.1 (10.9) | 35.6 (5.5) | n/a (-) |
| MBARI-M1 | 28.5 (5.5) | 15.2 (3.2) | 11.7 (1.5) | 23.9 (16.0) | 34.5 (13.2) | 32.5 (12.1) | 34.0 (7.1) | 27.1 (7.4) |
| NCOR-S1 | 27.8 (11.8) | 13.1 (4.3) | 13.5 (3.3) | 21.4 (5.7) | 36.0 (6.1) | 24.4 (8.1) | 26.8 (6.8) | 28.5 (12.9) |
| PAPA | 35.4 (15.1) | 14.8 (3.0) | 13.0 (3.1) | 24.9 (8.5) | 40.2 (8.2) | 39.9 (8.3) | 35.3 (8.2) | 30.6 (9.5) |
| NZOTS-STM | 39.5 (7.5) | 14.1 (3.2) | 15.5 (3.4) | 33.8 (10.6) | 25.5 (9.9) | 30.0 (13.0) | 23.7 (7.2) | 31.8 (9.7) |
| OOI-SOUTHERN | 47.5 (9.3) | 17.4 (7.2) | 23.0 (5.7) | 22.8 (12.1) | 30.1 (13.7) | 41.7 (11.5) | 43.8 (3.1) | 43.3 (11.3) |
| RATS | 43.0 (5.1) | 26.9 (9.1) | 11.8 (6.1) | n/a (-) | 28.0 (3.4) | 38.7 (12.4) | 42.7 (7.4) | 37.7 (14.7) |
| SOTS | 44.6 (10.5) | 20.1 (5.9) | 22.0 (8.4) | 30.3 (6.9) | 36.7 (12.8) | 45.8 (5.7) | n/a (-) | 37.8 (4.7) |

**Table S2:** Number of years of data required to detect a climate change-driven trend above background variability for 8 biogeochemical variables at BGC-SO sites. Values in brackets indicate standard deviation in *n** across the 8 model estimates. n/a indicates that the climate change trend does not exceed the natural variability in the timeframe of the simulations (95 years).

| **Footprint, 10^6^ km^2^** | **PP** | **SST** | **pH** | **Oxygen** | **Nitrate** | **Chlorophyll** | **Export** | **Non-diatom PP** |
| --- | --- | --- | --- | --- | --- | --- | --- | --- |
| BATS | 1.61 | 1.26 | 1.22 | 3.41 | 1.54 | 1.26 | 1.23 | 1.34 |
| CAPEBLANC | 0.56 | 1.45 | 1.57 | 0.27 | 0.91 | 0.54 | 0.54 | 0.66 |
| CVOO | 1.96 | 1.99 | 2.25 | 0.35 | 1.04 | 1.55 | 1.17 | 1.71 |
| ESTOC | 0.29 | 1.09 | 2.92 | 0.17 | 0.36 | 1.10 | 0.17 | 0.14 |
| K276 | 1.44 | 1.10 | 3.50 | 0.80 | 1.10 | 1.58 | 1.40 | 1.03 |
| OOI-ARGENTINE | 0.53 | 0.70 | 0.94 | 0.75 | 0.53 | 0.90 | 0.60 | 0.63 |
| OOI-IRMINGER | 1.58 | 1.48 | 0.48 | 1.69 | 1.66 | 1.48 | 1.28 | 2.00 |
| PIRATA 10W | 1.84 | 5.67 | 6.07 | 0.43 | 5.19 | 1.90 | 2.50 | 1.42 |
| PIRATA 38W | 1.81 | 3.38 | 2.70 | 0.58 | 1.37 | 1.25 | 1.57 | 1.52 |
| PAP | 1.47 | 1.12 | 1.69 | 1.43 | 1.11 | 1.69 | 1.58 | 1.67 |
| MIKE | 1.06 | 0.64 | 1.06 | 0.70 | 1.07 | 0.91 | 0.82 | 1.10 |
| A5 | 1.95 | 4.01 | 3.36 | 0.39 | 1.91 | 1.84 | 2.22 | 2.78 |
| A7 | 6.38 | 5.54 | 3.44 | 0.35 | 3.54 | 4.17 | 6.22 | 6.90 |
| ANTARES | 0.12 | 0.29 | 0.38 | 0.44 | 0.49 | 0.21 | 0.39 | 0.12 |
| DYFAMED | 0.26 | 0.05 | 0.07 | 0.31 | 0.06 | 0.30 | 0.21 | 0.19 |
| E1M3A | 1.22 | 0.74 | 0.70 | 0.55 | 0.84 | 1.57 | 1.33 | 0.93 |
| E2M3A | 0.08 | 0.09 | 0.09 | 0.70 | 0.28 | 0.13 | 0.08 | 0.04 |
| LION | 0.09 | 0.24 | 0.22 | 0.45 | 0.38 | 0.14 | 0.07 | 0.13 |
| CALCOFI-080-055 | 0.60 | 0.10 | 0.94 | 0.08 | 0.10 | 0.40 | 0.29 | 0.53 |
| CALCOFI-080-080 | 0.94 | 0.62 | 2.18 | 0.40 | 1.83 | 2.22 | 1.21 | 0.95 |
| CALCOFI-090-090 | 0.83 | 0.78 | 1.60 | 0.42 | 1.72 | 2.12 | 1.25 | 0.97 |
| ALOHA | 1.34 | 1.54 | 4.52 | 1.58 | 3.21 | 3.81 | 3.19 | 1.28 |
| K2 | 1.03 | 2.27 | 3.16 | 2.71 | 2.69 | 1.06 | 1.07 | 1.13 |
| JKEO | 1.05 | 1.45 | 1.19 | 0.54 | 1.99 | 0.62 | 0.87 | 0.60 |
| KEO | 1.46 | 1.46 | 1.49 | 0.63 | 1.32 | 1.02 | 0.73 | 1.39 |
| MBARI-M0 | 0.61 | 0.25 | 0.96 | 0.10 | 0.41 | 0.36 | 0.28 | 0.58 |
| MBARI-M1 | 0.60 | 0.26 | 1.42 | 0.25 | 0.35 | 0.10 | 0.85 | 0.43 |
| NCOR-S1 | 2.18 | 1.73 | 2.23 | 1.82 | 1.87 | 1.42 | 1.10 | 2.32 |
| PAPA | 2.53 | 2.06 | 3.12 | 0.99 | 4.67 | 2.48 | 2.09 | 2.33 |
| NZOTS-STM | 0.42 | 0.21 | 0.51 | 0.29 | 0.84 | 0.34 | 0.43 | 0.46 |
| OOI-SOUTHERN | 3.48 | 1.26 | 4.16 | 3.34 | 2.54 | 3.96 | 3.55 | 3.50 |
| RATS | 0.37 | 1.23 | 0.60 | 2.22 | 1.86 | 0.37 | 0.26 | 0.79 |
| SOTS | 2.44 | 1.27 | 2.78 | 2.99 | 1.73 | 4.19 | 1.80 | 2.80 |

**Table S3:** Size of footprints for all BGC-SOs (10^6^ km^2^) for all 8 variables.


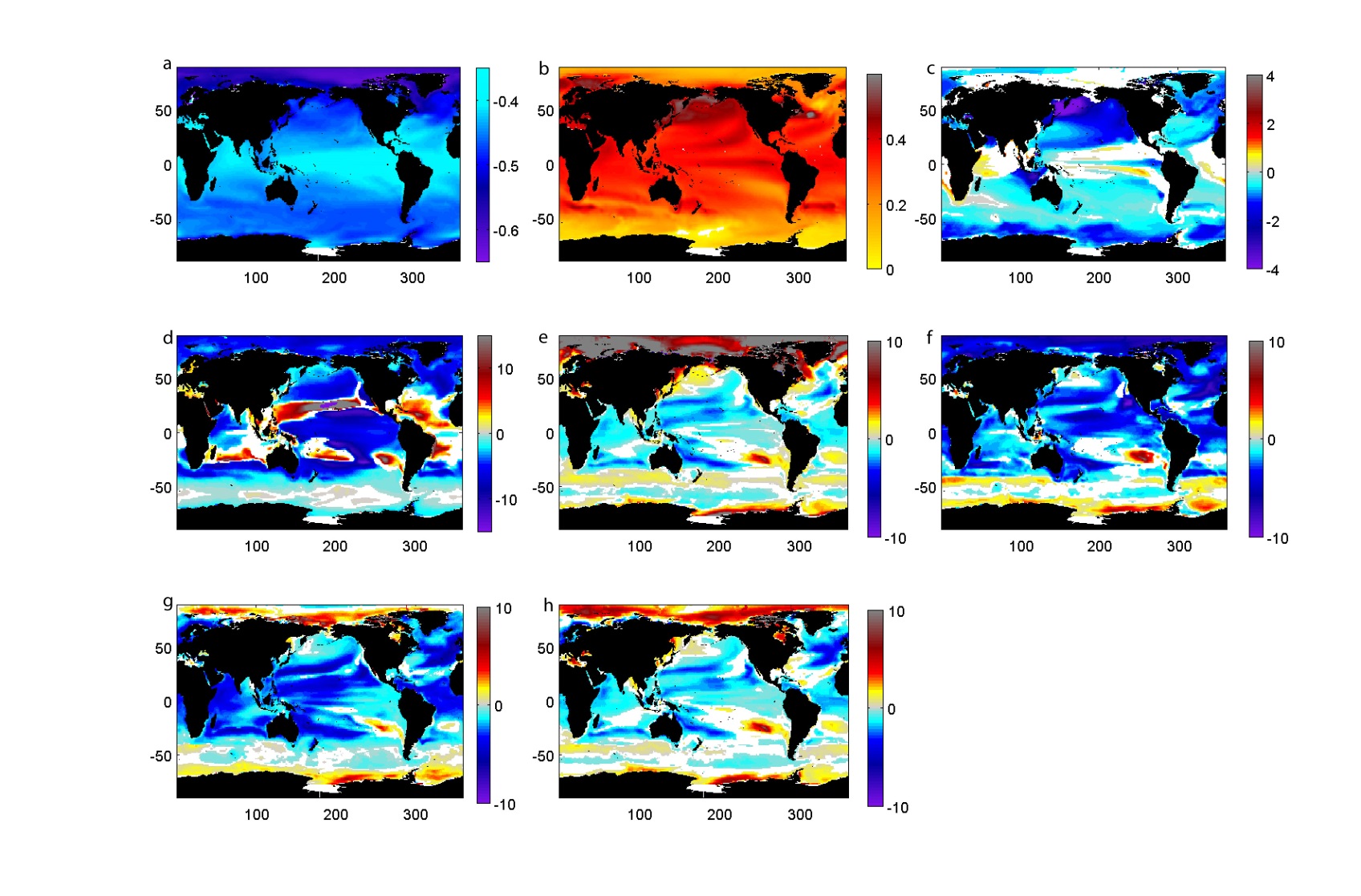


**Figure S1:** Trends in a) pH, b) SST, c) oxygen, d) nitrate, e) non-diatom PP, f) chlorophyll, g) export and h) PP, all expressed as % change per decade with respect to the mean of 1986-2005, except SST which is in °C per decade. White areas indicate where the regression is not statistically significant (p>0.05).


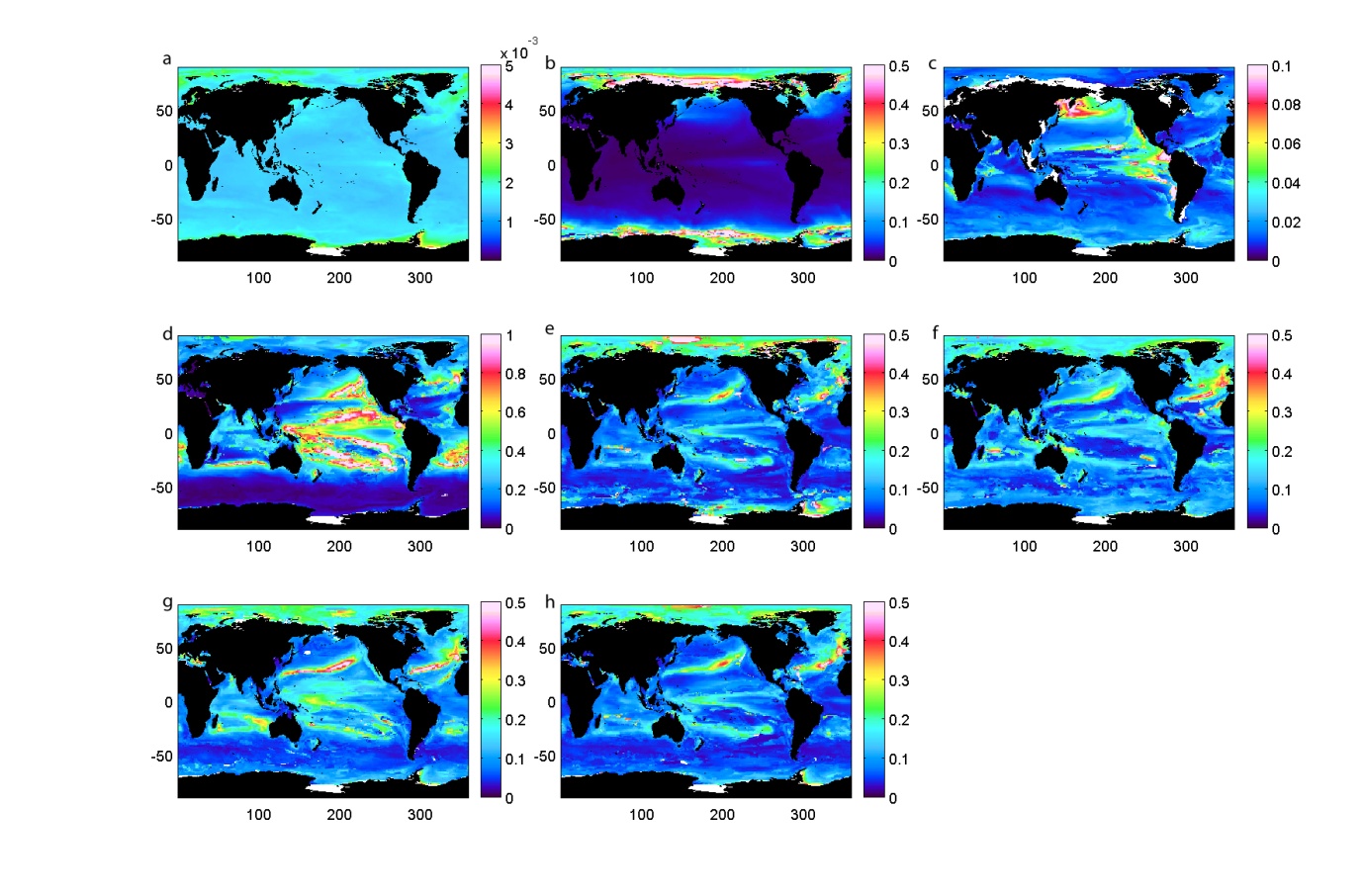


**Figure S2:**  Natural variability used in the calculation of *n** (equation 2) normalised to the model mean. The natural variability is the median of the standard deviation of the residuals divided by the model mean ($\sigma_{Nt}/\bar{Y_{t}}$) across all 8 models used here for a) pH, b) SST, c) oxygen, d) nitrate, e) non-diatom PP, f) chlorophyll, g) export and h) PP. Note different scales for different panels. Note that *n** is calculated independently for each model using each model’s corresponding control run.


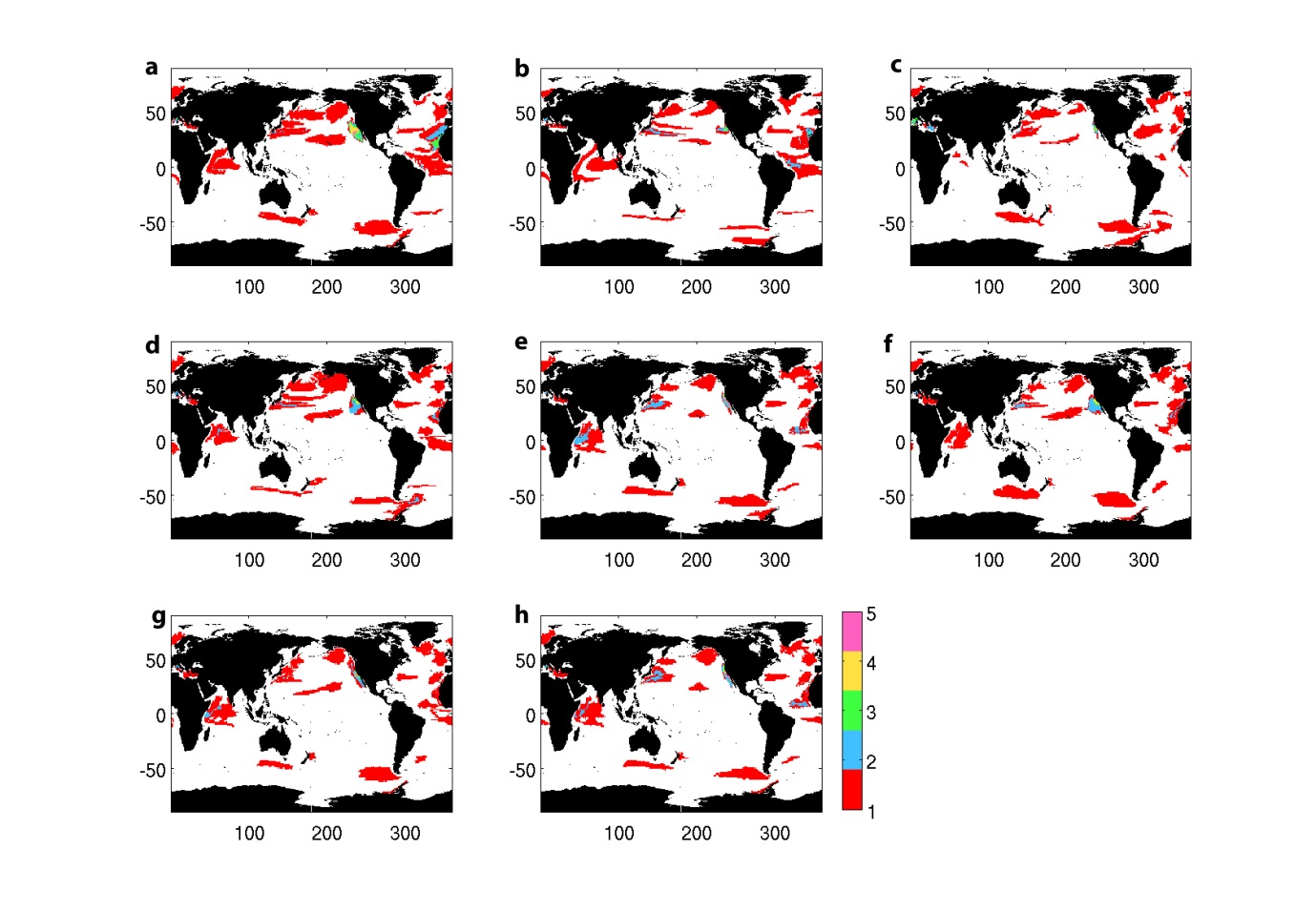


**Figure S3:** Number of BGC-SOs which have overlapping footprints, assessed pixel by pixel, for a) pH, b) SST, c) oxygen, d) nitrate, e) non-diatom PP, f) chlorophyll, g) export and h) PP. White indicates areas where no footprints occur and so are unrepresented by a BGC-SO.


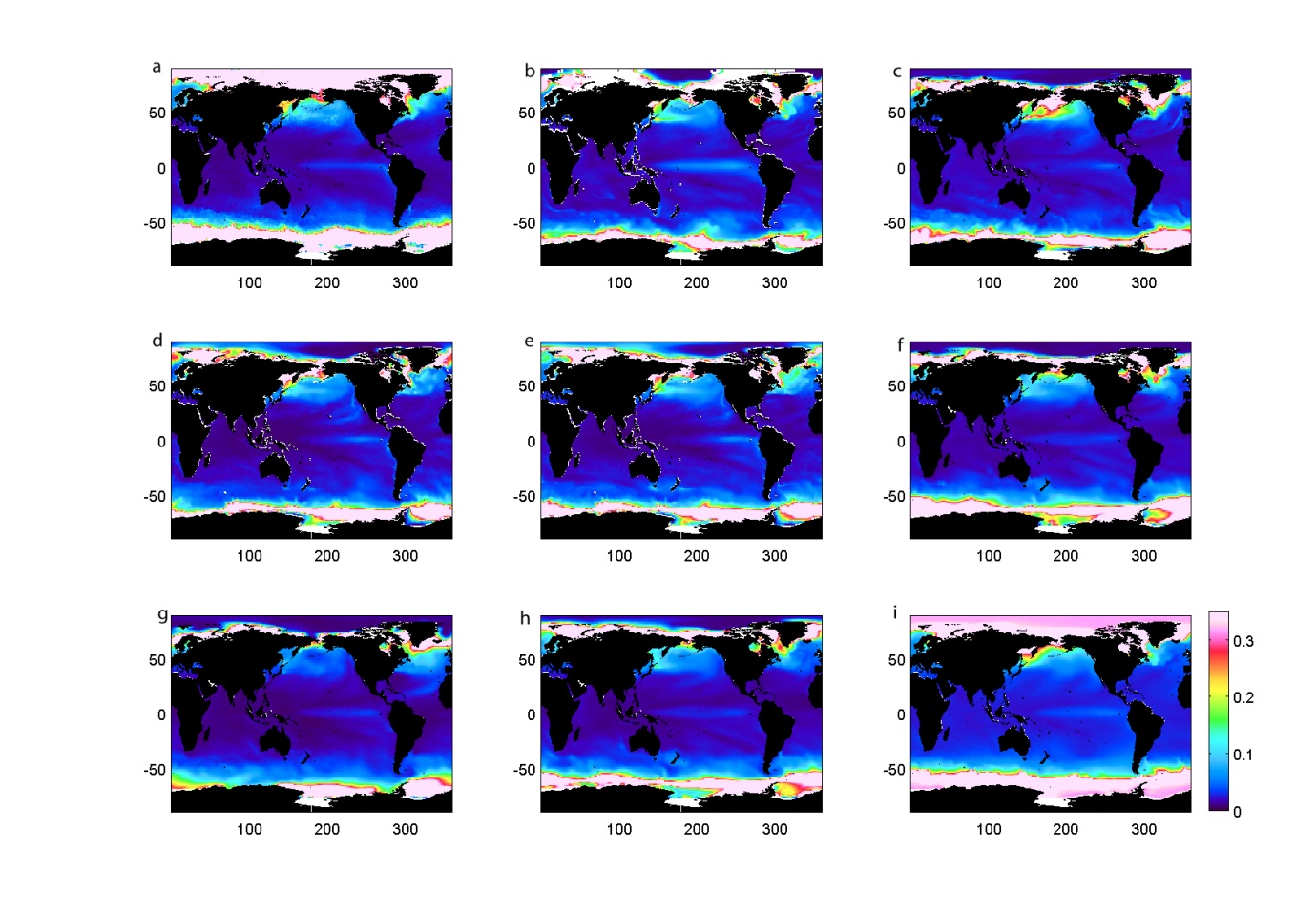


**Figure S4:** Comparison of interannual variability in SST in models used here with observations. Plotted is the coefficient of variation (standard deviation of annual mean SST/mean SST) calculated over the period of available satellite observations, 1985-2014. Panels show a) observations (AVHRR SST from 1985-2007 and MODIS-Aqua SST from 2008-2014), b) GFDL-ESM2M, c) GFDL-ESM2G, d) HadGEM2-CC, e) HadGEM2-ES, f) IPSL-CM5A-LR, g) IPSL-CM5B-LR, h) IPSL-CM5A-MR and i) CESM1(BGC).


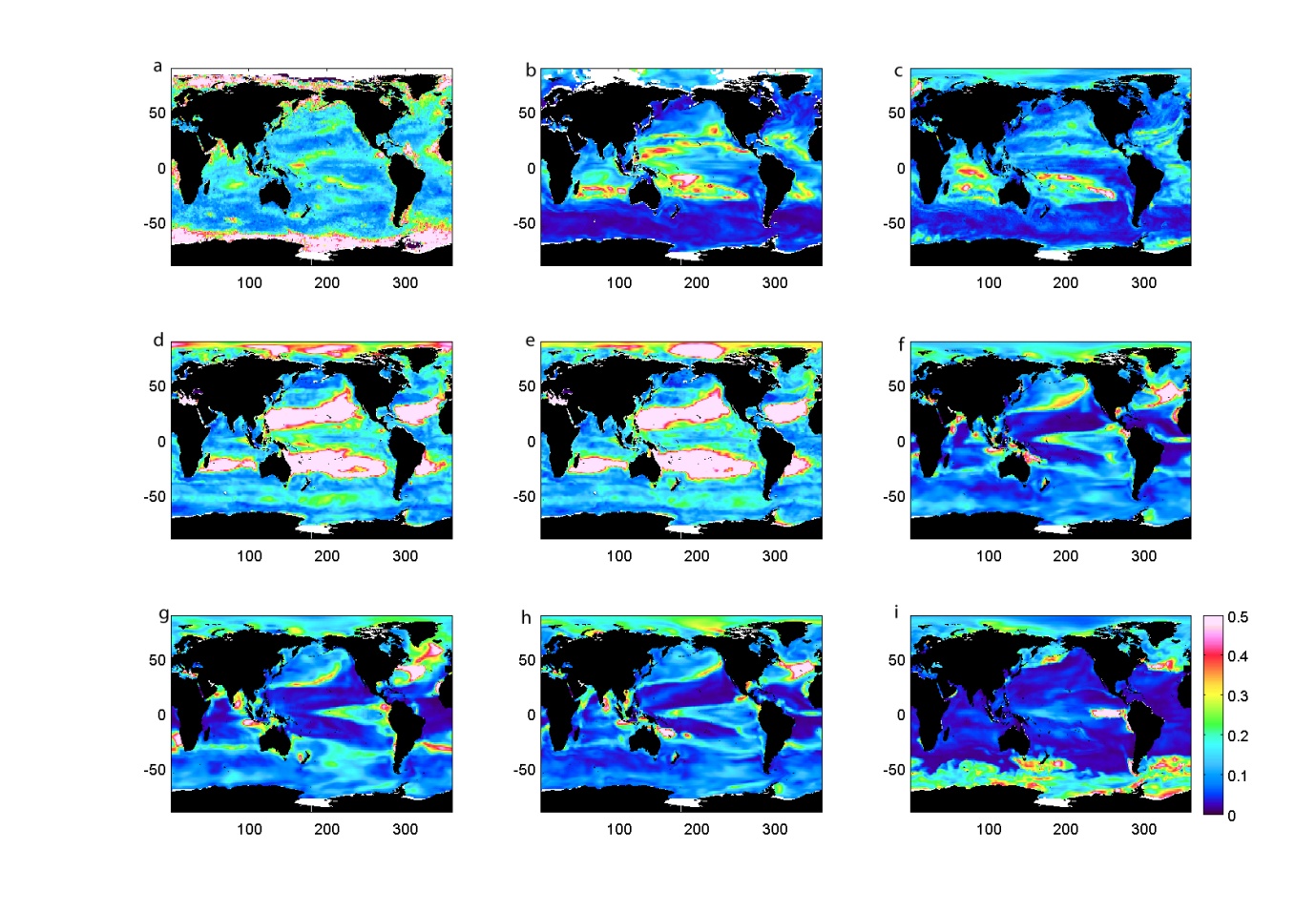


**Figure S5:** Comparison of interannual variability in chlorophyll concentration in models used here with observations. Plotted is the coefficient of variation (standard deviation of annual mean chl/mean chl) calculated over the period of available satellite observations, 1998-2014. Panels show a) observations (SeaWiFS chl from 1998-2007 and MODIS-Aqua chl from 2008-2014), b) GFDL-ESM2M, c) GFDL-ESM2G, d) HadGEM2-CC, e) HadGEM2-ES, f) IPSL-CM5A-LR, g) IPSL-CM5B-LR, h) IPSL-CM5A-MR and i) CESM1(BGC).


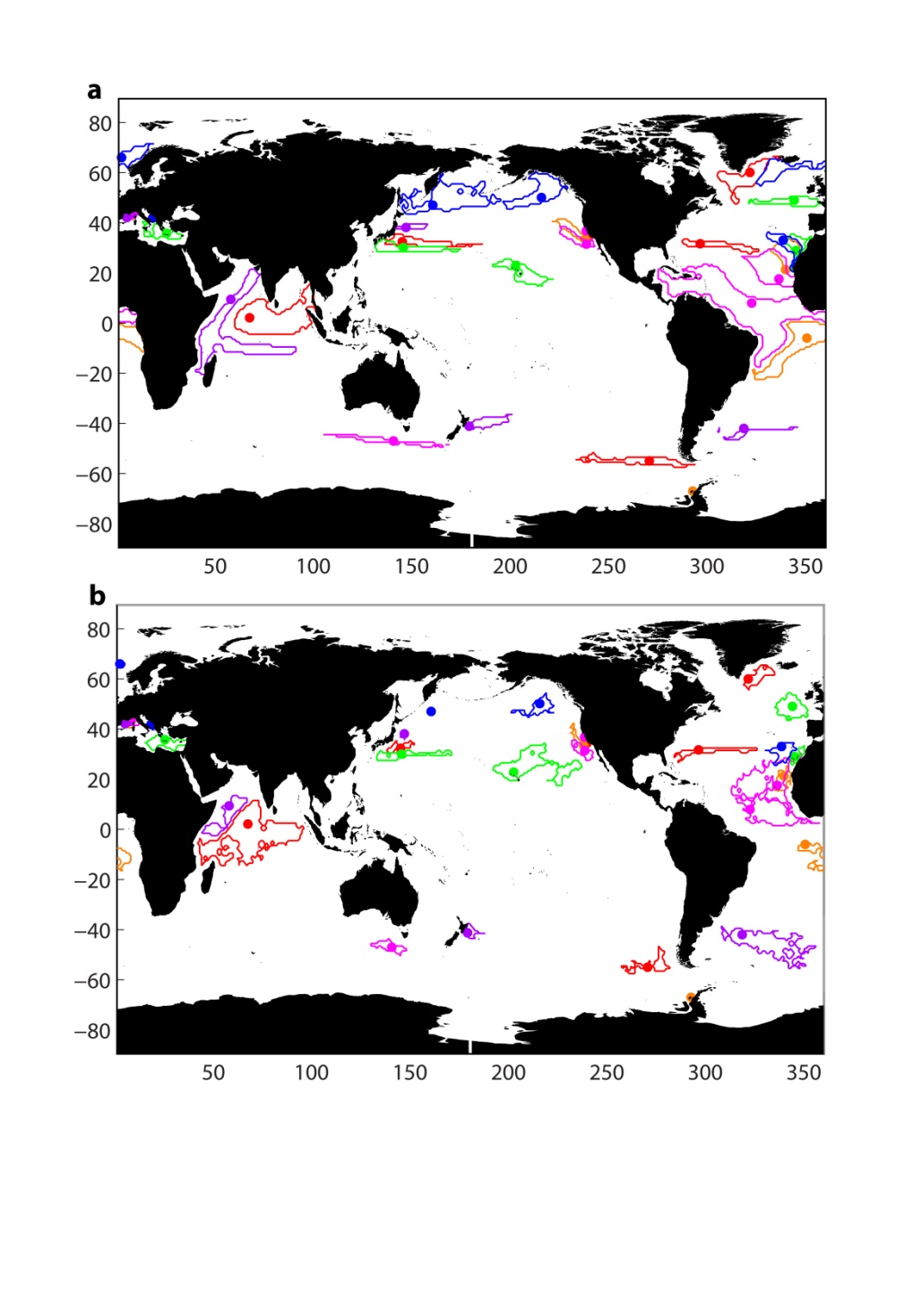


**Figure S6:** Spatial footprints calculated for a) SST and b) chlorophyll using satellite observations. SST is AVHRR data from 1985-2007 and MODIS-Aqua SST from 2008-2014; chlorophyll is SeaWiFS from 1998-2007 and MODIS-Aqua chlorophyll from 2008-2014. Equivalent maps for the ensemble model output can be found in Figure 3b and 3f.
